# Hybrid Zn-β-Aminoporphyrin–Carbon Nanotubes: Pyrrolidine and Direct Covalent Linkage Recognition, and Multiple-Photo Response

**DOI:** 10.3390/molecules28217438

**Published:** 2023-11-05

**Authors:** Susana L. H. Rebelo, César A. T. Laia, Monika Szefczyk, Alexandra Guedes, Ana M. G. Silva, Cristina Freire

**Affiliations:** 1 LAQV/REQUIMTE, Departamento de Química e Bioquímica, Faculdade de Ciências, Universidade do Porto, Rua do Campo Alegre s/n, 4169-007 Porto, Portugal; 2 LAQV/REQUIMTE, Departamento de Química, Faculdade de Ciências e Tecnologia, Universidade Nova de Lisboa, 2829-516 Caparica, Portugal; 3 Department of Bioorganic Chemistry, Faculty of Chemistry, Wroclaw University of Science and Technology, Wybrzeze Wyspianskiego 27, 50-370 Wroclaw, Poland; 4 Instituto de Ciências da Terra, Pólo da FCUP, Departamento de Geociências, Ambiente e Ordenamento do Território, Faculdade de Ciências, Universidade do Porto, Rua do Campo Alegre, 4169-007 Porto, Portugal

**Keywords:** zinc(II) porphyrin, carbon nanotube hybrids, photo-responsive materials, 1,3-dipolar cycloadditions, covalent linkage

## Abstract

To unveil and shape the molecular connectivity in (metallo)porphyrin–carbon nanotube hybrids are of main relevance for the multiple medicinal, photoelectronic, catalytic, and photocatalytic applications of these materials. Multi-walled carbon nanotubes (MWCNTs) were modified through 1,3-dipolar cycloaddition reactions with azomethine ylides generated in situ and carrying pentafluorophenyl groups, followed by immobilization of the β-amino-tetraphenylporphyrinate Zn(II). The functionalities were confirmed via XPS and FTIR, whereas Raman spectroscopy showed disruptions on the graphitic carbon nanotube surface upon both steps. The functionalization extension, measured via TGA mass loss and corroborated via XPS, was 0.2 mmol·g^−1^. Photophysical studies attest to the presence of the different porphyrin–carbon nanotube connectivity in the nanohybrid. Significantly different emission spectra and fluorescence anisotropy of 0.15–0.3 were observed upon variation of excitation wavelength. Vis-NIR absorption and flash photolysis experiments showed energy/charge transfer in the photoactivated nanohybrid. Moreover, evidence was found for direct reaction of amino groups with a carbon nanotube surface in the presence of molecular dipoles such as the zwitterionic sarcosine amino acid.

## 1. Introduction

Nanocomposites of porphyrins and carbon nanotubes are of great interest for a plethora of applications, owing to the unique and complementary properties of these two classes of compounds [1,2,3].

**Figure 1:**
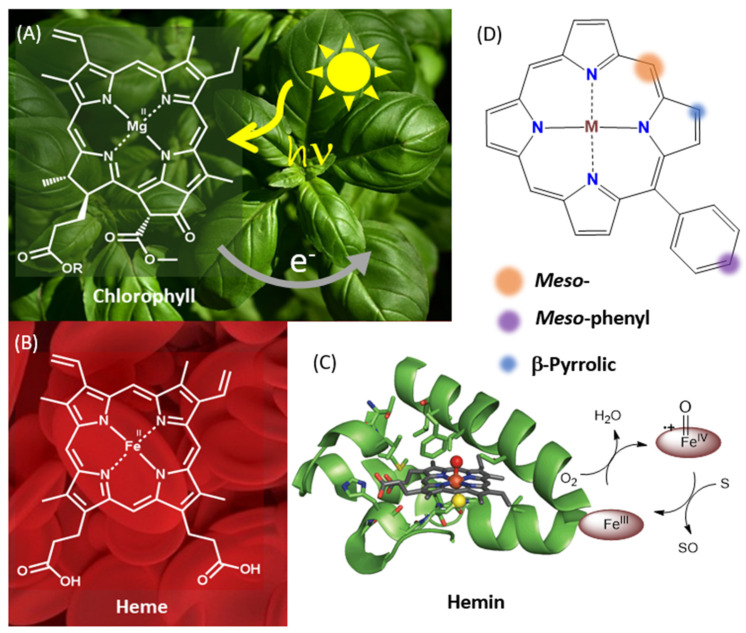
(**A**–**C**) Porphyrins in nature [4]; and (**D**) a synthetic analogous template with identification of porphyrin nucleus positions using different colors.

Porphyrinoid macrocycles have a prominent role in many biological functions, namely chlorophylls are solar light-harvesting antenna and initiators of the electron transport chain in photosynthesis (Figure 1A), while hemins are present in the active centers of many redox enzymes (Figure 1B,C) [4]. The biomimetic activity of synthetic metalloporphyrins (MPs) and metal-free porphyrins (H_2_P) has been amply explored for photoactivated and catalytic processes, with relevance in the development of sustainable processes [5,6].

Carbon nanotubes (CNTs) showed a cooperative action in diverse porphyrin (P) functions [7]. Diamagnetic MPs and the H_2_P are typically remarkable visible-light-responsive materials. However, they show poor electric conductivity and rapid charge recombination, which limit applications in the field of artificial photosynthesis, photocatalysis or photodynamic therapy (PDT) [8]. Hybrid materials MP/H_2_P-CNT have shown donor–acceptor behavior and effective charge separation for photo-induced electron transfer. These nanohybrids have promising applications in solar cells [9], photocatalytic oxidations [10], photo-generation of reactive oxygen species (ROS) [11], microbial photodynamic therapy for biomedical [12], and food applications [13]. MP-CNT hybrids also showed good performance in electrocatalysis for an oxygen reduction reaction (ORR) [14] and CO_2_ reduction reaction (CO_2_RR) [15], and in electroanalysis as bio-analyte sensors [16]. In the context of redox catalysis, MP-CNT hybrids showed efficient environmental pollutant oxidations [17] or as catalytic antioxidants, tailoring catalase/dismutase mimics [18].

The positive effect of CNT has been attributed to their high specific surface area, high electron density, redox properties, and high polarization of the graphitic surface [19]. CNTs also have a well-defined morphology and mechanical strength and, consequently, they show better batch-to-batch reproducibility when compared to other carbon supports, as graphene or activated carbons [17].

Typically, rigid multiwalled carbon nanotubes (MWCNTs) contrast with flexible single-walled carbon nanotubes (SWCNTs), while the latter show better conductivity. In photophysical processes, rigidity and conductivity are relevant. Furthermore, upon chemical functionalization, SWCNTs lose part of the initial conductive properties, while MWCNTs allow for chemical modifications on the outer shell but preserving most of the electronic properties, owing to the presence of inner shells [20]. For this reason, the hybrids of SWCNTs and porphyrins are often prepared via non-covalent functionalization, while numerous porphyrin–MWCNT materials were prepared via covalent linkages [15].

Previous works evidenced the effect of spacers on material photoactivity [21], as well as the linker position to the porphyrin nucleus [22]. Better performance was associated to β-pyrrolic relatively *meso*-phenyl linkages (Figure 1D) [23].

Efficient covalent strategies for CNT functionalization include oxidation and reactions with radicals or reactive dipolar species generated in situ [24]. Cycloaddition reactions to nanotube C=C bonds usually afforded a regular distribution of functional groups on the final material [25]. Instead, oxidative treatments are associated with open caps, nanotube shortening, disruption of the inner shells, and location of functional groups mainly at CNT edges [26]. Controllable catalytic processes are favored by uniform distributions of the functional groups on the material.

**Figure 2:**
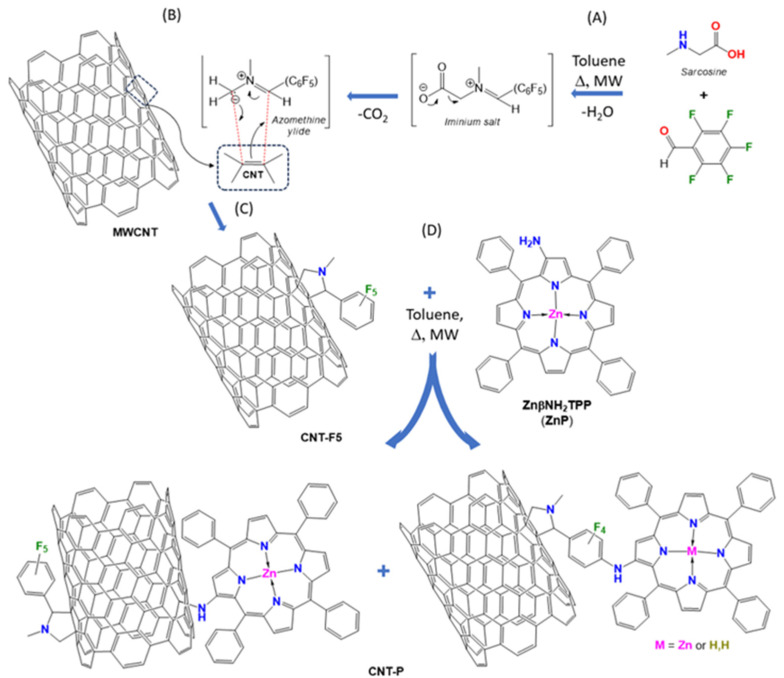
Preparation of Znporphyrin–carbon nanotubes hybrid (CNT-P): (**A**–**C**) 1,3-cycloaddition reaction by pentafluorophenyl–azomethine ylides generated in situ affording material CNT-F5. (**D**) Possible pathways for Znporphyrin immobilization.

The present work reports the immobilization of an electron donor Zn(II) porphyrin on MWCNT through a β-linkage (Figure 2) in two steps. The functionalization of CNT sidewalls with electron withdrawing pentafluorophenyl–pyrrolidine groups was carried out through a 1,3-dipolar cycloaddition reaction with azomethine ylides formed in situ (Figure 2A,B), followed by nucleophilic substitution of the pentafluorophenyl ring with the Zinc(II) complex of β-aminotetraphenylporphyrin (ZnβNH_2_TPP, ZnP). However, other connectivity can be obtained due to the inherent reactivity of the CNT surface [27].

Physicochemical characterization of the nanohybrid included a comprehensive XPS study aimed to obtain information on the modifications of the CNT surface and relate the molecular linkages to the photophysical properties.

## 2. Results and Discussion

### 2.1. Functionalization of Carbon Nanotubes

In a first step, MWCNTs were modified by azomethine ylides carrying a pentafluoro-phenyl group, leading to the material CNT-F5. Both DMF and toluene have been tested as the solvents (Figure 2A–C). The condensation of sarcosine with pentafluorobenzaldehyde and thermal decarboxylation of the resulting iminium salts affords azomethine ylides [28], which undergo 1,3-dipolar cycloaddition reactions with the CNT double bonds, which work as dipolarophiles (Figure 2B).

In the second step, material CNT-F5 reacted with Zn(II) β-amino-tetraphenylporphyrinate (ZnβNH_2_TPP, ZnP) in toluene as the solvent, to produce the carbon nanotube–porphyrin nanohybrid (CNT-P, Figure 2D). Both reactions have been carried out under microwave heating.

An expected reaction path is the nucleophilic substitution on the 4-position F-atom of the pentafluorophenyl ring by the NH_2_–porphyrin to afford the CNT-P1 moieties (Figure 2D). Still, since the direct reaction of CNT with anilines has been described when dipolar species are present [26,27], in parallel, the reaction of the amino-porphyrin and CNT may occur, whereas CNT-P2 moieties might also be expected.

### 2.2. Physicochemical Characterization

#### 2.2.1. XPS Data and Analysis

X-ray photoelectron spectroscopy (XPS) analysis was carried out for CNT materials and the atomic percentages are displayed in Table 1. The XPS survey spectra of the materials are presented in Supplementary Material (Figure S1). Pristine MWCNTs (CNTs) show high C purity, showing O atomic percentage ≤ 2%, and N was not detected (Table 1, entry 1).

The functionalization of CNTs by azomethine ylides was confirmed by the presence of N and F in material CNT-F5 (Table 1, entry 2). This reaction was tested both in toluene and DMF. Despite the latter led to better dispersion of the nanotubes, a lower F/N ratio was obtained (Table 1, entry 3 versus entry 2), probably due to nucleophilic substitution of the *p*-F-atoms of the pentafluorophenyl ring by dimethylamine arising from DMF decomposition at a reflux temperature [29]. Considering that the diameter of the carbon nanotubes used (<10 nm) corresponds to the penetration of the X-ray beam in the XPS analysis area, the extent of metalloporphyrin immobilization on the CNT was calculated based on the at% of Zn in the nanohybrid CNT-P (0.2 %), indicating the ZnP (M = 693 g·mol^−1^) anchoring with a loading of 0.16 mmol·g^−1^.

**Figure 3:**
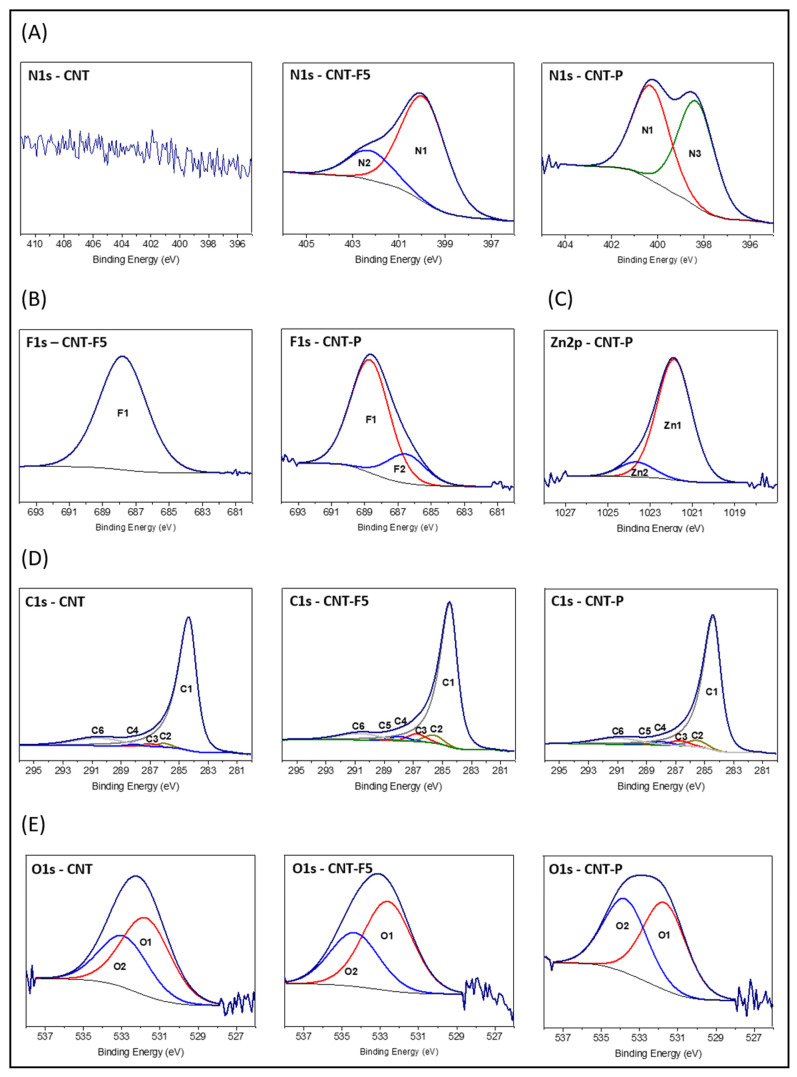
Curve-fitting of the high-resolution XPS spectra from CNT materials in the element regions N1s (**A**), F1s (**B**), Zn2p (**C**), C1s (**D**) and O1s (**E**).

High-resolution XPS spectra in the detected elemental regions have been a valuable tool for gaining a deeper understanding of the functional groups and molecular connections on the materials’ surfaces [27]. The spectra of CNT materials are shown in Figure 3. The curve-fitting was carried out using the methodologies described in the Supplementary Materials, and the obtained components and their assignments are shown in Table 2.

Regarding the N1s spectra (Figure 3A), although this element is not observed in pristine MWCNT, it is detected in material CNT-F5 upon azomethine ylide functionalization. Two components are present, the N1 at ~400.2 eV that is mainly ascribed to pyrrolidine groups and the N2 at 402.2 eV that is primarily attributed to cationic (protonated) N. This latter can be explained by the presence of sarcosine easily adsorbed on the dipole-induced nanotube surface [30]. Previous density functional theory studies support the strong adsorption of zwitterionic glycine onto a carbon nanotube surface [31], whereas protonation of the tertiary amine groups in an azomethine ring is less likely.

For material CNT-P, upon ZnβNH_2_TPP immobilization, the N1s spectrum shows components N1 and N3; the latter at 498.3 eV is ascribed to pyrrolic N from the porphyrin core [32]. Non-detection of the N2 component might be explained by leaching of the aa upon the second functionalization step.

The F1s spectrum of material CNT-F5 (Figure 3B) shows a single band at ~688 eV ascribed to aromatic F (Ar-F). The atomic percentage ratio of the components F1/N1 is in accordance with the presence of pentafluorophenyl–pyrrolidine rings.

On the other hand, the F1s spectra of nanohybrid CNT-P show two components. F1 corresponds to Ar-F moieties and F2, at 686.5 eV, ascribed to F^−^, which probably results from the nucleophilic substitution reaction (Figure 2D), is retained at the CNT surface. The F2 component (20%) attests to an extensive *p*-substitution on the pentafluorophenyl ring (Figure 2D).

The Zn2p spectrum of material CNT-P shows two significantly different chemical environments for Zn (Figure 3C), Zn1 at 1021.9 eV typical of Zn(II) cations, and Zn2 at 1023.7 eV. The latter was observed previously on a nanohybrid through direct ZnP–CNT linkage [23] (Figure 2D, CNT-P2 that is further supported by the photophysical experiments presented in Section 2.3).

The curve-fitting of C1s spectra was obtained with five components for original CNTs and six components for functionalized materials (Figure 3D). The components were assigned using an oxygenated CNT material as the reference [33]: C1 at ~284.5 eV ascribed to C=C; C2 at 285.5–286.1 eV to C-C; C3 at ~287 eV to C-O; C4 at ~288.1 eV to C=O; C5 at ~289.1 eV to COO^−^ (from sarcosine) and C6 at ~290.8 eV to π-π* stacking.

Original MWCNTs show a very small percentage of C2, C3, and C4 components, being ascribed to C-C, C-O, and C=O bonds, respectively, from deposition of adventitious carbon, which has been attributed to short chain carbon with small amounts of both singly and doubly bound oxygen functionalities and from CNT-OH groups due to residual oxidations. This matches the O1s spectra, showing 2 at% of O (Figure 3E).

Table 1 shows that component C1, and concomitantly component C6, decreases upon CNT reaction with azomethine ylides (Figure 2A). This is in accordance with the conversion of graphitic C=C bonds into pyrrolidine rings, which also accounts for the increase in C-C bonds percentage in material CNT-F5, as is observed by Raman spectroscopy (Section 2.2.4). On the other hand, nanohybrid CNT-P (Figure 1D) shows a new increase in C1 and C6 percentages, in accordance with the introduction of C=C bonds from porphyrin macrocycles.

In the present curve fitting, C-C/C-H bonds are observed at 286.1 eV in original CNT (1.6%) and at ~285.5 eV for functionalized materials (~3.6%, Table 1), which might indicate a different type of C-C (defects) introduced upon functionalization [34].

Higher percentages of C3 and C4 and the appearance of C5 are observed upon functionalization in materials CNT-F5 and CNT-P. In material CNT-F5, the C-N and C-F bounds overlap C3 and explain the higher C3% (4.3%), while *p*-(C*-F)-(C-F)n bounds add to C4% due to *p*-carbons that cumulate adjacent C-F bond effects [35]. In addition, the eventual presence of adsorbed sarcosine contributes to C4 (C-N^+^ bounds) and to C5 (COO^−^ groups).

In material CNT-P, although the absence of sarcosine was deduced from the N1s spectrum, the percentages of C4 and C5 are nearly identical to those of CNT-F5 (~2% and ~0.5%, respectively). This can be explained by imine bonds (C=N) from the porphyrin nucleus that overlap C4 and protonated imine bonds (C=N^+^-H) that overlap C5 [32]. These results support the presence on the CNT of the ZnP and indicate a small fraction of protonated porphyrin [H_4_TPP]^2−^ (see fluorescence studies in Section 2.3).

The O1s spectra (Figure 3E) show two components: O1 at 531.7–532.6 eV ascribed to C=O and O2 at 532.9–534.2 eV ascribed to C-O. Even though in the CNT-F5 material the component BEs shift from the original CNT, the proportion of the two components remains the same, which is in line with the introduction of the COO^−^ group from sarcosine, which contributes equally to the two components. In the material CNT-P, the relative % of component O2 increases, probably due to chemisorbed water.

#### 2.2.2. FTIR Data and Analysis

**Figure 4:**
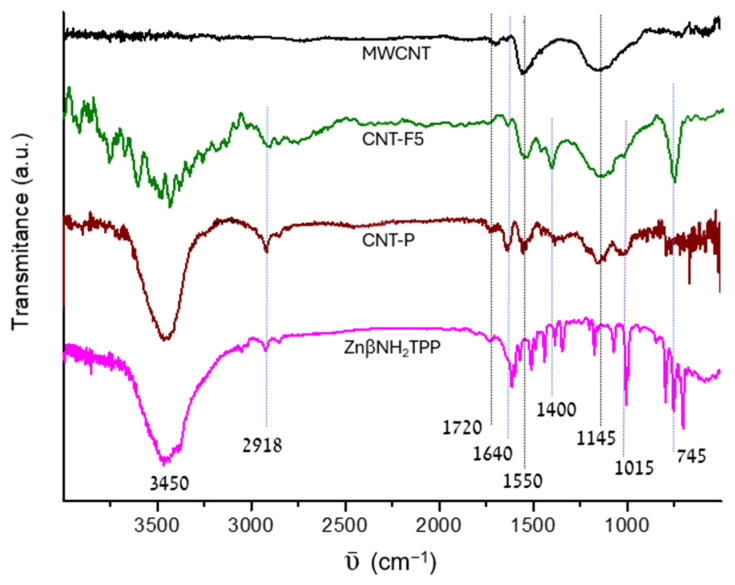
FTIR spectra of the original MWCNT and functionalized materials CNT-F5 and CNT-P in comparison with porphyrin ZnβNH_2_TPP.

The Fourier transform infrared spectroscopy (FTIR) measurements of the CNT show low-intensity and broad bands (Figure 4) that are typical in graphitic materials. Graphite has no FTIR spectrum, and the observed signals are ascribed to functional groups introduced or to defects on the graphitic structure [23].

A pristine MWCNT shows a band at ~1550 cm^−1^ and a shoulder at ~1650 cm^−1^, which are attributed to C=C bonds near the defects, present in small aromatic domains or alkene bonds, respectively. The broad band in the 1000–1200 cm^−1^ region is ascribed to skeletal C-C tangential motions, being also observed in other carbon materials. It probably has a contribution from C-O bonds, whereas the small band at 1700 cm^−1^ is ascribed to residual C=O.

Upon azomethine ylide functionalization, a relatively intense band at ~745 cm^−1^ is observed in material CNT-F5, which is ascribed to the C-F stretching from the pentafluoro phenyl rings, while the bands due to ν_C-N_ can be overlapped by the band at ~1100 cm^−1^. The presence of C-H bonds is shown by the signals at ~2910 and ~2850 cm^−1^ (ν), and at ~1400 cm^−1^ (δ). Moreover, an increase in the relative intensity of the band at 1650 cm^−1^ is associated with the covalent functionalization of the graphitic surface with loss of aromaticity [27]. The intense band at ~3450 cm^−1^ is due to ν_O-H_ and ν_N-H_.

The FTIR spectrum of material CNT-P also confirms the success of the porphyrin immobilization reaction. In the CNT-P spectrum, an intensification or broadening of the bands from CNT-F5 is observed due to overlapping of the bands present in the ZnβNH_2_TPP spectrum, namely, the intensification of the bands at 1015 cm^−1^, 1640 cm^−1^, 2920 cm^−1^, and 2850 cm^−1^ ascribed to porphyrin vibrations ν_C-N_, ν_C=N_/ν_C=C_, and ν_C-H_, or the broadening of the bands at 750 cm^−1^ and 1400 cm^−1^.

#### 2.2.3. Thermogravimetric Analysis

**Figure 5:**
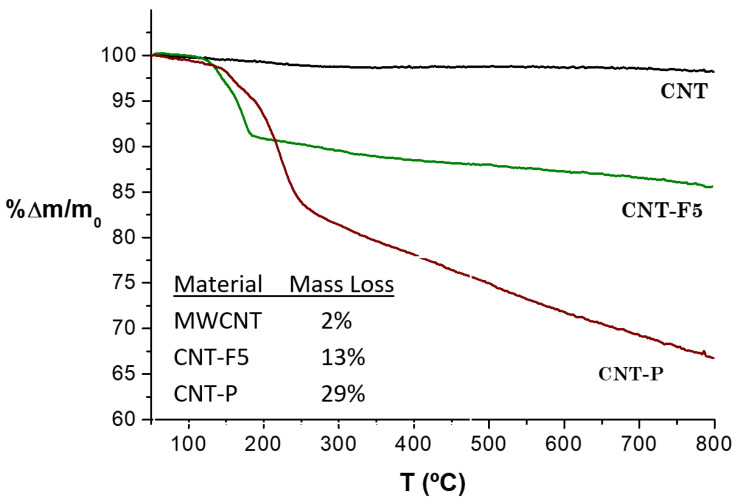
Thermograms of MWCNTs and functionalized materials. The inset shows the total mass losses (%) observed for the materials.

The thermogravimetric analyses (TGA) attest to the thermic stability of a pristine MWCNT until 800 °C, and confirm their good graphitic quality (Figure 5). The small mass loss % in the temperature range of 100–800 °C is explained by the decomposition of residual oxygenated surface groups, which corroborate the XPS at% and FTIR results presented above. Consequently, for materials CNT-F5 and CNT-P, the total mass loss can be related to the decomposition of surface-attached moieties, and this indicates the functionalization extent.

For material CNT-F5, the pronounced mass loss in the 200–300 °C range is attributed to the decomposition of the pyrrolidine groups and indicates a homogeneous functionalization of these groups in the material [29].

The difference in mass loss between the CNT-P and CNT-F5 materials gives an indication of the % of mass introduced upon ZnP functionalization reaction of 16 wt%. The shape of the TGA curve for material CNT-P in the temperature range of 250–800 °C is in line with that typically observed for tetraphenylporphyrins [29]. Attending to the molar mass of the ZnβNH_2_TPP (M = 693 g/mol), a mass loss of 16 wt% corresponds to a porphyrin loading of 0.2 mmol·g^−1^, in accordance with the value obtained via XPS.

#### 2.2.4. Raman Spectroscopy

**Figure 6:**
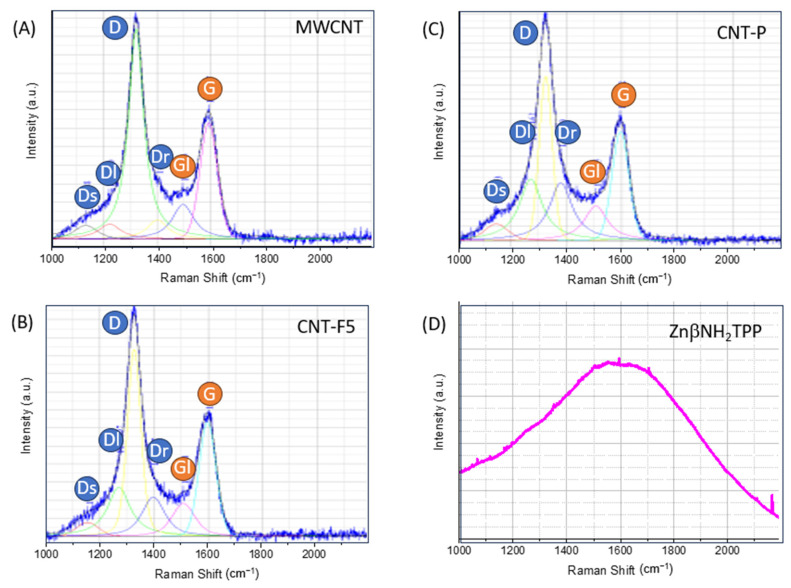
Raman spectra of CNT materials (**A**–**C**) and ZnβNH_2_TPP (**D**). Blue circles mark the D bands and orange circles mark the G bands.

The curve-fitting and interpretation of the Raman spectra were based on a multi-band model and considered in the first-order region four “D bands” and two “G bands” [26]. The D band in the Raman spectra is typically assigned to disruptions (disorder) in the graphite structure. However, in MWCNT, the deconvolution of this band needs to distinguish disorder associated to curvature and interaction between the layers, from the disorder related to the disruption of the surface graphite sheet by covalent immobilization, which is expected to be observed in the present materials (Figure 6, deconvolution criteria in the Supplementary Materials).

Raman studies of polycyclic aromatic hydrocarbons (PAHs) show bands at near 1250 and/or 1400 cm^−1^, while polyacetylene shows two features at 1200 and 1500 cm^−1^. Thus, the bands Dl, Dr, and Gr were assigned to the presence of low-size aromatic domains, PAH analogues, while the DS and Gl bands were assigned to alkenes and conjugated alkenes. Furthermore, the Raman spectra of the fullerenes show a base feature at 1500 cm^−1^, and the Gl band also has contributions from the curvature and tube-end vibrations [26].

The cycloaddition reactions are expected to disrupt the graphitic surface of MWCNT, creating PAH-like domains; thus, it is expected that upon functionalization, an increase in the relative intensity of the Dl and Dr bands is observed relative to the bands related to the graphitic nanotube structure, i.e., D and G [26]. Table 3 shows the ratios of the band intensities for Dl+Dr/D and Dl+Dr/G. As the intensity of the Gl band also does not change upon functionalization, the Dl+Dr/G ration was calculated as well.

The results confirm a significant loss of graphitic conjugation due to covalent functionalization of material CNT-F5 upon reaction with azomethine ylides, and that a relatively smaller loss of graphitic conjugation also occurs upon ZnβNH_2_TPP immobilization. These observations give support for the direct reaction of aminoporphyrin with the CNT surface occurring to a small extent. The porphyrin spectrum (Figure 6D) covers the whole spectral range, but does not influence the CNT-P spectrum, as the intensity of the G band only changes slightly.

### 2.3. Photophysical Studies

**Figure 7:**
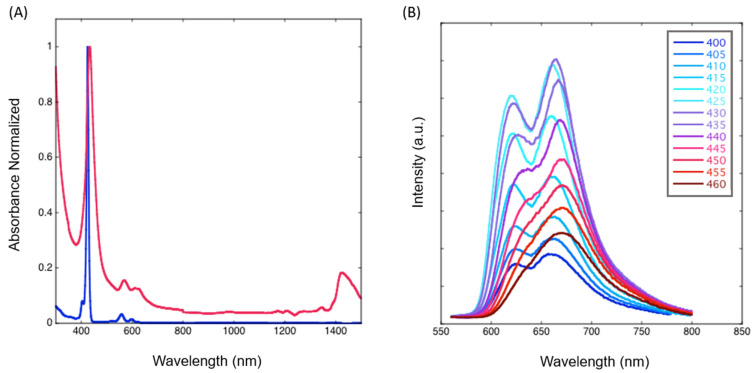
(**A**) Absorption spectra in the VIS/NIR range of ZnβNH_2_TPP (blue) and CNT-P (red) in DMF. (**B**) Emission spectra of the CNT-P changing the excitation wavelength.

The absorption spectra of CNT-P in the DMF dispersions were recorded in the Vis/NIR region and compared with the ZnβNH_2_TPP spectrum (Figure 7A). The Soret and Q bands are clearly broader for CNT-P. Moreover, the wavelength of the maximum absorption of Soret/Q bands of CNT-P at 433/570 nm is red-shifted relative to those of ZnP at 425/559 nm. Broadening and red shifting are indicative of changes in the electronic states of the macrocycle and/or energy/electronic coupling between the porphyrin and CNT π-systems, and suggest the presence of bonds/interactions between them. An absorption band appears at 1425 nm, which is attributed to CNT. Also, the CNT-P sample displays light scattering throughout the entire wavelength range that has features like Rayleigh scattering.

The CNT-P emission was studied by changing the excitation wavelength (Figure 7B). The spectra show great changes in Q emission bands changing the excitation wavelength in the 400–460 nm range, indicating the existence of multiple ZnP species that interact in a different manner with the CNT. Excitation of the nanohybrid at 425 nm (ZnβNH_2_TPP Soret) shows the reverse intensities of the Q bands relative to ZnβNH_2_TPP [23] (Supplementary Material, Figure S2A). This indicates that leaching of physically adsorbed ZnβNH_2_TPP to CNT is probably not being observed.

Three types of emission patterns can be distinguished in this study: a predominant one resulting from excitation at around 425 nm, while two other patterns give rise to less intense spectra. Upon excitation from 400 to 415 nm, there is an increase in the relative intensity of the emission band on the left at 621 nm that then decreases for excitations at higher wavelengths, probably due to the residual presence of a different chromophore with the Soret band circa 415 nm. This can be ascribed to the residual presence of immobilized metal-free porphyrin since Zn removal can occur at relatively low proton concentrations.

Another clearly different emission spectral shape was obtained by excitation at around 460 nm (a single band was obtained at around 675 nm). This latter spectral shape is analogous to the one described for the fluorescence of a material prepared by direct binding between ZnP and CNTs [23]. Its presence is also corroborated by the Zn high-resolution XPS spectra (Figure 3C).

**Figure 8:**
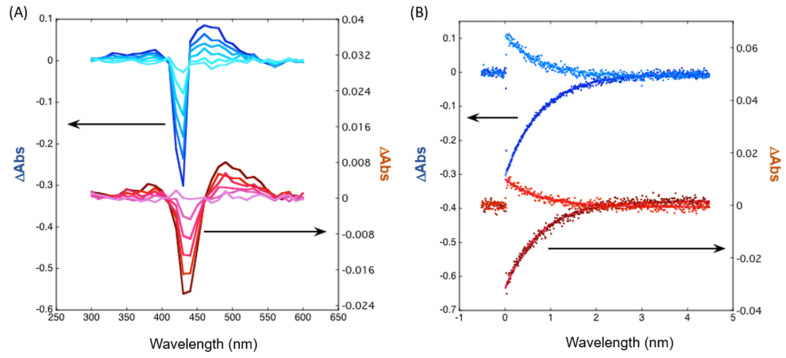
Transient spectroscopy: (**A**) Time-resolved absorption spectra of ZnβNH_2_TPP (blue) and CNT-P (red) in aerated DMF (time delays 0.3, 0.5, 0.7, 1.0, 1.5, and 2.5 μs and λ_ex_ = 355 nm; (**B**) Flash photolysis kinetic traces of ZnβNH_2_TPP (blue) and CNT-P (red) in DMF (fitting with single exponential with lifetime of 0.7 μs, same conditions as in (**A**).

Laser flash photolysis experiments also show the broadening of the Soret band in the transition absorption spectra, as well as a marked decrease in the transient absorption signal for CNT-P (Figure 8A). Moreover, different absorption features for CNT-P relative to ZnβNH_2_TPP (Supplementary Material, Figure S2B), namely at 350, 490, and 530 nm, also indicate the presence of the differences in the macrocycles present in the nanohybrid (transient absorption τ = 0.278 μs).

The triplet lifetime in aerated media is identical for CNT-P and ZnβNH_2_TPP, and triplet decays remain single-exponential (Figure 8B) (7 μs). Thus, the efficiency of porphyrin triplet quenching by oxygen remains identical, while energy/charge transfer to CNT might increase other pathways for ROS formation other than singlet oxygen, such as the superoxide radical or hydroxyl radical pathways.

**Figure 9:**
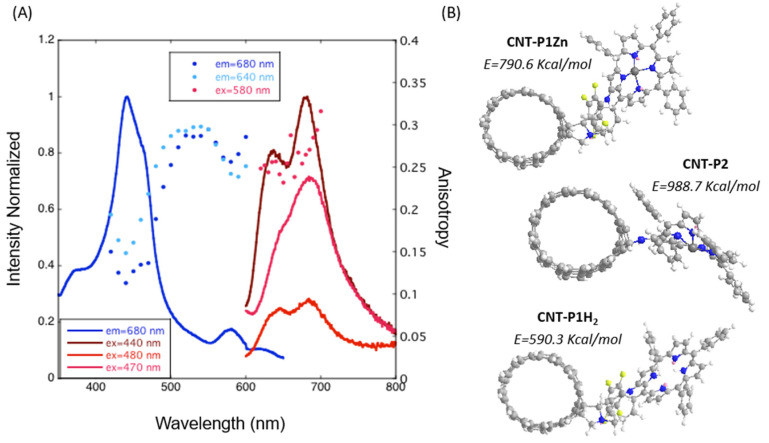
(**A**) Emission and excitation spectra of CNT-P in an ionic liquid (Aliquat Chloride) and fluorescence anisotropy. (**B**) Energy calculated for energy minimization for the three proposed chromophores on CNT-P (E—total energy).

Emission spectroscopy in an ionic liquid (Figure 9) also reveals dependence with excitation wavelength. Fluorescence anisotropy, however, reveals additional features in the interaction between ZnβNH_2_TPP and CNT. The excitation anisotropy values change from about 0.15 when excited in the Soret band, to about 0.3 when going to the Q bands, which also gives rise to a change in the emission shape. High values of anisotropy indicate short fluorescence lifetimes and/or immobilization of the emitting molecule. Thus, we conclude that a different mobility is present in the attached porphyrins, probably by two different types of covalent bonds, with one leading to a more hampered porphyrin macrocycle (Figure 2D, CNT-P).

Figure 9B shows the minimum energies obtained in Chem-3D (ChemOffice) for representations of the three proposed chromophores attached to a CNT (representing the curved graphitic external sheet of the MWCNTs). These corroborate a less hindered structure for the linkage through the pentafluorophenyl–pyrrolidine linker than that through direct amine linkage. The lowest energy was observed for the immobilized metal-free porphyrin.

### 2.4. Parallel Functionalization

Previous work demonstrated that anilines establishing isomeric equilibria with stabilized dipolar species (such as aminophenol) can directly react with carbon nanotubes [27]. The mechanism was ascribed to the further stabilization of the dipolar intermediate species by electrostatic interactions with the carbon nanotube wall, with characteristic electronic delocalization and charge migration, followed by an attack of nucleophiles on a reactive (δ^+^) carbon. An analogous mechanism may justify the present results and formation of CNT-P2 (Figure 2D). Zwitterionic sarcosine might be present and work as a dipole-inducing molecule in the CNT surface, allowing for the attack of the ZnβNH_2_TPP as a nucleophile and the porphyrin anchoring.

Other nucleophiles present in the reaction media might also react in a similar way, such as F^−^ and water. The latter was also observed in the previously reported direct reaction between CNT and substituted anilines [27].

Furthermore, the direct reaction of ZnβNH_2_TPP with MWCNTs in the absence of additives, such as sarcosine or a radical initiator, is described in literature and no functionalization was observed [23].

## 3. Materials and Methods

### 3.1. Chemicals

All reagents and solvents were reagent-grade and used as received. MWCNTs were obtained from Nanocyl S.A., Namur, Belgium, ref ^a^ NC3100 thin MWCNT, with an average diameter of 9.5 nm, 1.5 µm length, and purity > 95% C.

### 3.2. Functionalization of CNTs

#### 3.2.1. Pentafluorophenyl-*N*-Methylpyrrolidine Carbon Nanotubes (CNT-F5)

In a 50 mL microwave reaction vessel, MWCNTs (200 mg) were dispersed in the reaction solvent (DMF or toluene, 40 mL), and sarcosine (9 mmol, 0.8 g) and pentafluorobenzaldehyde (4.5 mmol, 0.88 g) were added. The suspension was de-aerated under nitrogen flow, capped, and sonicated for 5 min. The vessel was introduced in the microwave apparatus and heated to 140 °C (DMF) or 110 °C (toluene), setting the maximum pressure to 200 psi for 1 h. The addition of sarcosine and pentafluorobenzaldehyde, de-aeration, sonication, and heating procedures were repeated four more times. The total amounts added were 0.044 mol (4 g) for sarcosine and 0.0225 mol (4.4 g) for pentafluorobenzaldehyde. At the end of the reaction, the CNTs were filtered under vacuum through a polyamide membrane (0.2 µm), rinsed successively with methanol and ethanol, and finally dried at 110 °C overnight.

#### 3.2.2. Preparation of Nanohybrid (CNT-P)

The ZnβNH_2_TPP was prepared using a previously described procedure, by reduction in the parent nitro-porphyrin with H_2_ in the presence of a Pd/C catalyst [36]. In a 50 mL microwave reaction vessel, the material CNT-F5 (100 mg) was dispersed in 40 mL of toluene, and sonicated for 5 min. Then, 25 mg of ZnβNH_2_TPP was added and the reaction mixture de-aerated and heated in the microwave oven for 1 h. After cooling, the material was filtered through a polyamide membrane of 0.2 µm under vacuum, and extensively washed with ethanol. The resulting material was dried in the oven at 110 °C overnight.

### 3.3. Physico-Chemical Characterization

X-ray photoelectron spectroscopy (XPS) was performed at “Centro de Materiais da Universidade do Porto” (CEMUP, Porto, Portugal) in a VG Scientific ESCALAB 200A spectrometer (Thermo Scientific, Walthan, MA, USA) using non-monochromatized Al Kα radiation (1486.6 eV). The powdered samples were pressed into pellets prior to the XPS studies. The binding energies were calibrated relative to the C1s peak at 284.6 eV. The XPS spectra were deconvoluted by curve-fitting peak components using the software CasaXPS (version 2.3.19PR1.0, Casa Software Ltd., Teignmouth, UK) with no preliminary smoothing. Symmetric Gaussian–Lorentzian product functions were used to approximate the line shapes of the fitting components after a Shirley-type background subtraction (criteria used in the deconvolution of the element high-resolution spectra are described in the Supplementary Materials section). The atomic % was calculated from the experimental intensity in the high-resolution spectra, and normalized by atomic sensitivity factors.

Fourier transform infrared spectra (FTIR) of the materials were collected with a Jasco FTIR-460 Plus spectrophotometer (JASCO corporation, Tokyo, Japan) in the 400−4000 cm^−1^ range, using a resolution of 4 cm^−1^ and 32 scans. The spectra of the samples were obtained in KBr pellets containing 0.2 wt% MWCNT.

Raman spectra were recorded directly on the materials using a LabRAM Dilor–Jobin-Yvon–Spex spectrometer (Horiba, Kyoto, Japan), with the monochromatic red light at 632.8 nm of the He–Ne laser as the excitation source, at a power of 20 mW. Adequate fits to the experimental data were obtained using a mixed Gaussian–Lorentzian curve-fitting procedure in a Labspec program. The fitting was performed without fixing or limiting the range of any spectral parameter in the iteration procedure.

Thermogravimetric analysis (TGA) was carried out on a STA 409 PC device (NETZSCH, Selb, Germany). All samples (typically 5–7 mg) were heated under nitrogen flow (200 cm^3^.min^−1^) to 800 °C at a rate of 10 °C·min^−1^.

Photophysical characterization: Absorption spectra were acquired on a Shimadzu UV-2501PC (Shimadzu Corporation, Kyoto, Japan) or a CARY 100Bio (Varian, Palo Alto, CA, USA), and fluorescence spectra were obtained on a Jobin Yvon Spex, Fluorolog FL3-22 (Horiba, Kyoto, Japan). Nanosecond laser flash photolysis experiments were run on a LKS.60 nanosecond laser photolysis spectrometer from Applied Photophysics (Leatherhead, UK).

The 3D structures and minimum energy calculations were performed in Chem3D, version 20.0.0.41 (PerkinElmer, Waltham, MA, USA).

## 4. Conclusions

An environmentally compatible and two-step functionalization of MWCNTs with pentafluorophenyl pyrrolidine groups, followed by aminoporphyrin immobilization, led to a highly functional and photo-responsive material. Photophysical and high-resolution XPS results attest to the presence of different covalent linkages between the Zn(II) porphyrin and carbon nanotubes assigned to the expected pyrrolidine–pentafluorophenyl–amino spacer and a direct amino linkage. The later corroborated and expanded previous studies that reported the direct functionalization of CNTs with anilines in the presence of dipolar molecules and in the absence of a radical initiator. The third chromophore observed was assigned to the presence of a metal-free immobilized porphyrin fraction.

## Figures and Tables

**Table 1:** Surface atomic percentages of the elements calculated from XPS.

		At %					
Entry	Material	C1s	N1s	O1s	F1s	Zn2p3	F/N
1	CNT	98.0	<DL ^a^	2.0	-	-	-
2	CNT-F5	92.4	1.2	1.5	4.9	-	4.1
3	CNT-F5_DMF_^b^	93.4	1.5	2.0	3.1	-	2.1
4	CNT-P	95.5	1.0	1.25	2.0	0.2	2.0

^a^ Under the detection limit; ^b^ This material was prepared through a reaction in DMF as the solvent instead of toluene used in the other reactions.

**Table 2:** Binding energies and relative area percentages of the components obtained via curve-fitting of core-level XPS spectra of relevant elements in the nanomaterials.

**Material**	**BE, eV (Relative Area, %) ^a^**							
	**C1s**						**O1s**	
	**C1**	**C2**	**C3**	**C4**	**C5**	**C6**	**O1**	**O2**
CNT	284.4 (85.9)	286.1 (1.6)	287.1 (1.3)	288.2 (0.8)	-	290.7 (10.5)	531.7 (60.2)	532.9 (39.2)
CNT-F5	284.5(82.6)	285.6 (3.5)	286.7 (4.3)	288.0 (2.2)	289.0 (0.5)	290.7 (6.8)	532.6 (62.4)	534.0 (37.6)
CNT-P	284.4(83.5)	285.5 (3.7)	286.7 (2.7)	288.0 (1.9)	289.2 (0.6)	290.9 (7.6)	531.7 (50.5)	533.8 (49.5)
	C=C	C-C, C-H	C-O, C-N, C-F	C=O, C=N, C-N^+^, (C-F)_n_	COO, C=N^+^	π to π ^b^	C=O, (C=O)-O	C-OH (C=O)-O
**Material**	**BE, eV (Relative Area, %) ^a^**							
	**N1s**				**F1s**		**Zn2p**	
	**N1**	**N2**	**N3**		**F1**	**F2**	**Zn1**	**Zn2**
CNT	-	-	-		-	-	-	-
CNT-F5	400.1 (72.7)	402.2 (27.3)	-		687.8 (100)	-	-	-
CNT-P	400.3 (47.2)	-	398.3 (52.8)		688.7 (79.5)	686.5 (20.5)	1021.9 (89)	1023.7 (11)
	N-H	N^+^-C	N=C		Ar-F	F^−^	Zn-P1	Zn-P2

^a^ Percentage of each component area relative to the total core-level peak area; ^b^ Aromatic shake-up satellite peak.

**Table 3:** Significant ratios from the Raman spectral bands of the CNT materials.

Material	Dl+Dr/D	Dl+Dr/Gl	Dl+Dr/G
MWCNT	0.12	0.8	0.21
CNT-F5	0.39	2.6	0.72
CNT-P	0.46	3.1	0.84

## Data Availability

Data are contained within the article and supplementary materials.

## Supplementary Materials

The following supporting information can be downloaded at: https://www.mdpi.com/article/10.3390/molecules28217438/s1, Figure S1: XPS survey spectra of materials in Table 1: (A) original CNT; (B) CNT-F5; (C) CNT-F5_DMF_; (D) CNT-P; Figure S2: Photophysical comparison of ZnβNH_2_TPP (blue) and CNT-P (red) in DMF: (A) Emission spectra; (B) Time-resolved absorption spectra; Figure S3: Complete data and different structure perspective for minimization energy calculations for the three proposed chromophores on CNT-P.
