# Supplementary material for: Hybrid Zn-β-Aminoporphyrin–Carbon Nanotubes: Pyrrolidine and Direct Covalent Linkage Recognition, and Multiple-Photo Response
Source: Molecules. 2023 Nov 5;28(21):7438. doi: 10.3390/molecules28217438 (PMC10649226; doi:10.3390/molecules28217438)
Supplement: Supplementary file 1 [file molecules-28-07438-s001.zip › molecules-2642047-supplementary.pdf]

Supplementary Material

Hybrid Zn-β-Aminoporphyrin–Carbon Nanotubes: Pyrrolidine and Direct Covalent Linkage Recognition, and Multiple-Photo Response

Susana L.H. Rebelo,[a*] Cesar A.T. Laia,[b] Monika E. Szefczyk,[a,c] Alexandra Guedes,[d] Ana M.G. Silva,[a] Cristina Freire[a]

[a] LAQV/REQUIMTE, Departamento de Química e Bioquímica, Faculdade de Ciências, Universidade do Porto, Rua do Campo Alegre s/n, 4169-007 Porto, Portugal

[b] LAQV/REQUIMTE, Departamento de Química, Faculdade de Ciências e Tecnologia, Universidade Nova de Lisboa, 2829-516 Caparica, Portugal

[c] Wroclaw University of Science & Technology, Faculty of Chemistry, Department of Bioorganic Chemistry, Wybrzeie Wyspianskiego 27, PL-50370 Wroclaw, Poland

[d] Instituto de Ciências da Terra, Pólo da FCUP and Departamento de Geociências, Ambiente e Ordenamento do Território, Faculdade de Ciências, Universidade do Porto, Rua do Campo Alegre, 4169-007 Porto, Portugal

*Corresponding author - E-mail: Susana.rebelo@fc.up.pt

Details on analytical techniques

XPS studies

High-resolution XPS spectra deconvolutions were obtained with the following definitions and constrains:

Shirly type background subtraction.

Deconvolution of C1s spectra was obtained with asymmetric line shapes for graphitic C=C bonds LA(1.2,2.3,0) for pristine MWCNT and LA(1.2,2.1,0) for functional materials. All other bands were deconvoluted with a GL(30) line shape. Bands C2-C5 were constrained to the same FWHM. In the final fitting, the % of carbon bonds [sum of components: C1(C=C), C2(C-C and C-H) and C6(π-π*)] matches the value of C% obtained for the sample. For pristine MWCNT the sum of percentage of components C3 and C4 matches the value of O% obtained for the sample.

The deconvolution of the high-resolution spectra in the regions of O1s, N1s, F1s and Zn2p was carried out using a GL(30) line shape with constrains to the same FWHM.

Raman spectroscopy

Once the D band is a resonant mode and exhibits dispersive behavior, both its position and shape can vary with different excitation laser frequencies. Therefore, the excitation laser frequency was maintained for all measurements. A density filter was used to avoid thermal decomposition of samples by the laser. The 100x objective lens of an Olympus optical microscope was used to focus the laser beam on the sample and to collect the scattered radiation. A highly sensitive CCD camera was used to collect the Raman spectra. Each Raman spectrum presented in this study corresponds to the accumulation of 20 spectra recorded from 1000 to 3500 cm^{-1} over 30 s; five accumulated spectra were collected for each sample to access the within-sample heterogeneity, at spectral resolutions near 1 cm^{-1}.

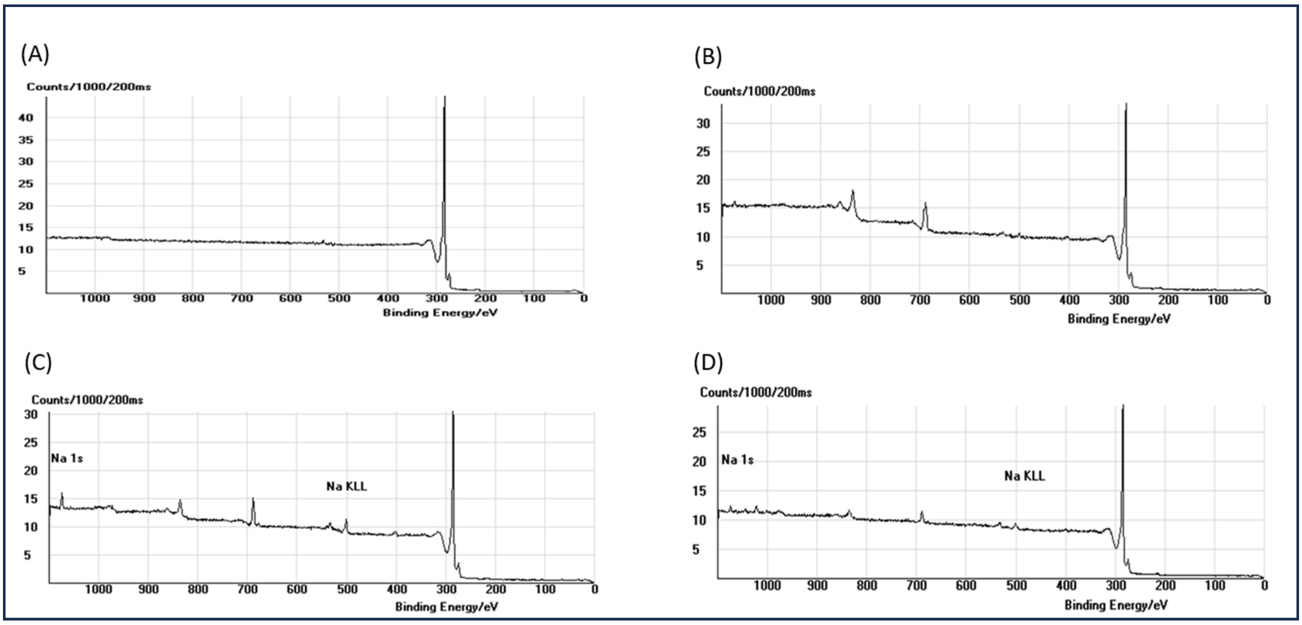

Figure S1. XPS survey spectra of materials in Table 1: (A) original CNT; (B) CNT-F5; (C) CNT-F5$_{DMF}$; (D) CNT-P.

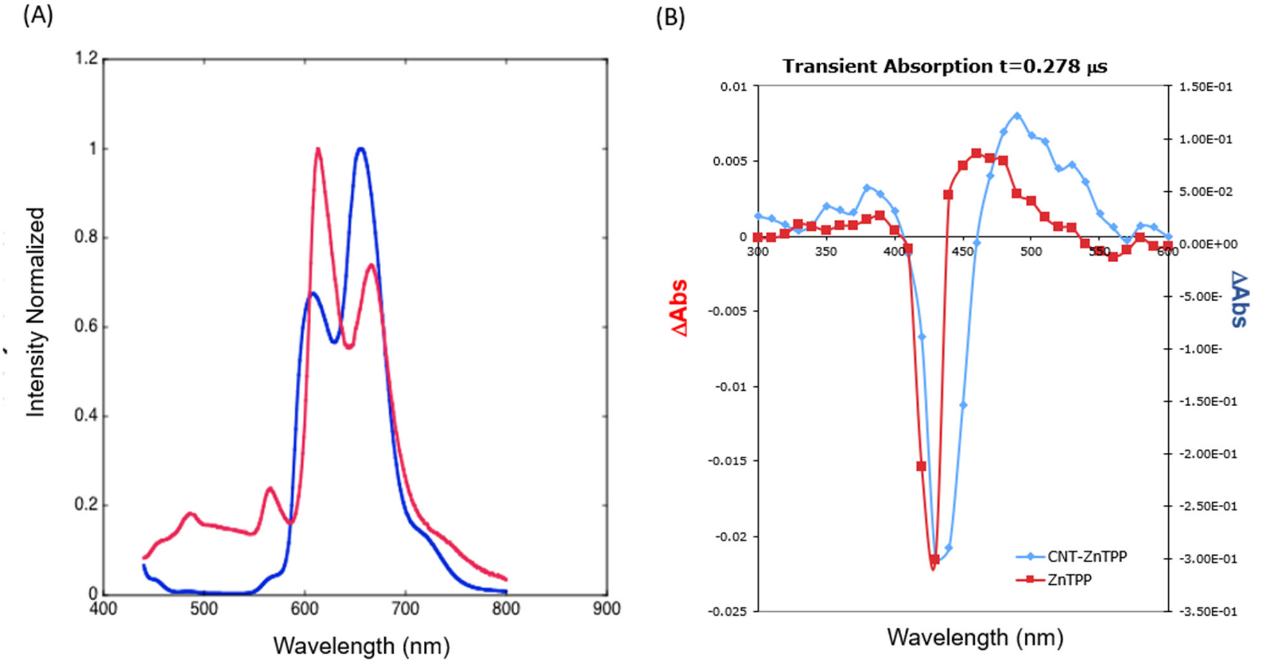

Figure S2. Photophysical comparison of ZnβNH$_2$TPP (blue) and CNT-P (red) in DMF: (A) Emission spectra; B) Time-resolved absorption spectra.

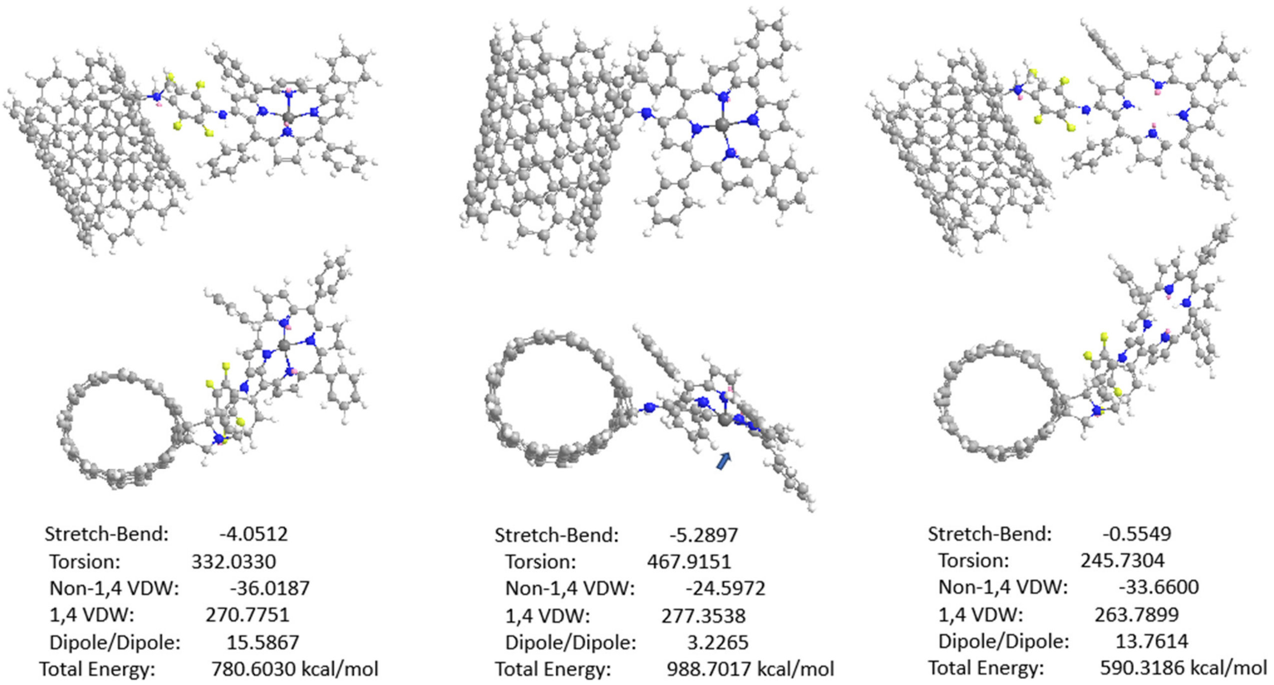

Figure S3. Complete data and different structure perspective for minimization energy calculations for the three proposed chromophores on CNT-P.
